# The association between weather and the number of daily shootings in Chicago (2012–2016)

**DOI:** 10.1186/s40621-020-00260-3

**Published:** 2020-06-22

**Authors:** Paul M. Reeping, David Hemenway

**Affiliations:** 1 Department of Epidemiology, Mailman School of Public Health, Columbia University, 722 West 168th Street, New York, NY 10032 USA; 2 Department of Health Policy and Management, Harvard T. H. Chan School of Public Health, 677 Huntington Avenue, Kresge Building Room 309, Boston, MA 02115 USA

**Keywords:** Firearms, Gun violence, Urban violence, Weather, Epidemiology

## Abstract

**Background:**

Previous studies have linked weather to crime and aggression but have not considered the causal structure of the variables included in the model(s).

**Methods:**

This cross-sectional study used data from 2012 to 2016 to measure the association between weather and the number of shootings in Chicago. The number of shootings per day was obtained via the Chicago Tribune (2012–2016). Daily high temperature, humidity, wind speed, difference in temperature from historical average, precipitation type and amount, were extracted via The Weather Underground. Weekend, holidays, and other non-school days were also included as possible effect measure modifiers. Causally-adjusted negative binomial regressions were used to evaluate the associations between the exposures of interest and daily number of shootings.

**Results:**

A 10-degree (°C) higher temperature was significantly associated with 34% more shootings on weekdays, and 42% more shootings on weekends or holidays. A 10-degree higher temperature than average was also associated with 33.8% higher rate of shootings.

**Conclusion:**

In recent years, shootings in Chicago were more likely to happen on warm days and especially during the weekend or holidays. This finding is in-line with studies that have linked crime to higher temperature and also suggests that shootings may be related to when individuals are outside and more likely to encounter violence. Interventions that keep people inside, such as air-conditioning and summer programs for students, might be effective in reducing the number of shootings in Chicago. We believe using a causal structure is useful for understanding the link between weather and shootings.

## Background

Chicago is often perceived as one of the most violent cities in the United States; in 2016, there were over 4100 victims of gun violence, or the equivalent to one individual being shot every 2 hours (Crime in Chicago, 2018). Chicago had a homicide rate of 16.4 per 10,000 citizens and a non-fatal firearm violent injury rate of 88.9 per 10,000 in 2015, respectively ranking Chicago 18th and 14th among cities in the nation (Hertz, 2014). Most of the gun violence in Chicago occurs in specific geographic locations and can be associated with socioeconomic factors and race. For example, areas in the north, which are wealthier and more white (Wilson & Daly, 1997), have as few as 0–5 annual homicides per 10,000 citizens, while neighborhoods in the south and west side have over 35 annual homicides per 10,000 (Mirabile, 2016). The most affected group--young, adult, Black males-- have a 1 in 200 yearly chance of being shot (Papachristos et al., 2015).

While there has been a substantial amount of research about the micro-locations where shootings are most likely to occur, there has been less on when shootings occur (Block & Block, 1995; Eck et al., 2005). One way to predict when shootings will occur is by looking at weather. For example, a review found that weather, including temperature and precipitation, was associated with conflict in Mexico, India, Tanzania, Australia, and the Philippines (Burke et al., 2015). Other studies have found that as temperature increased aggression also increased (Baron & Ransberger, 1978; Anderson et al., 2000; Anderson, 1987; Kenrick & MacFarlane, 1986; Cotton, 1986; Field, 1992; Anderson, 1989). Most recently, investigators in Baltimore found that precipitation was associated with a decrease in violent crime, and higher temperature was associated with an increase in firearm shootings and violent crime (Michel et al., 2016). However, these studies were predictive rather than causal and it is not appropriate to interpret the coefficients as stand-alone factors. For example, do thunderstorms predict shootings? Or do shootings occur more often when it is warm, and high temperature increases the probability of a thunderstorm? To be able to interpret each coefficient, the complex causal structure of each variable must be considered.

One prior study examined the relationship between weather and shootings in Chicago (Kieltyka et al., 2016). The authors found that as temperature increased, the number of daily shootings in Chicago also increased, and rain significantly decreased the number of firearm related injuries and crime. We update that study for a more recent period in Chicago when shootings were increasing rapidly, using what we believe are more complete and valid data (e.g., their study missed homicides going directly to the morgue and the injured either not seeking medical attention or leaving the hospital within 2 hours), and an established causal structure that goes beyond prediction, allowing us to make recommendations for policy.

Our cross-sectional study used publicly available data from 2012 to 2016 to measure the association between 10 ecological variables and the number of shootings in Chicago, after considering the complex causal structure between all of the variables and possible effect measure modifiers. We hypothesize that weather and other situations that lead to more people being outside will be associated with more shootings.

## Methods

### Data sources and variables

For our cross-sectional study, we used the Chicago Tribune website (2012–2016) to obtain a daily count of shooting victims (injured or killed) in Chicago (Crime in Chicago, 2018). A team at the Chicago Tribune Breaking News Desk searches all news reports for instances of interpersonal injury or homicide caused by a firearm. We used this media-based data source instead of hospital records because those who die of a gunshot wound before reaching a trauma center, or who are admitted to the hospital but die or are discharged within 12 h, are not recorded in Chicago hospital databases. By contrast, the Chicago Tribune obtains data on any individual who is shot, regardless of whether they go to the hospital, die, or refuse treatment.

We used the Weather Underground as a source for historical weather-related variables (Weather History for KORD, 2018) measured at Troposphere Group Station KILCHICA658. Maximum daily temperature (°C), maximum daily relative humidity (%), maximum daily wind speed (kph), precipitation type (categorical: snow, rain, or thunderstorm, reference = no precipitation), precipitation amount (cm), and historical temperature averages (°C) were included in this analysis. Difference in historical temperature average (°C) was obtained by subtracting the daily maximum temperature from the historical maximum average temperature (the average temperature on that date between 1930 and 2020).

We examined other ecological variables not related to weather. Holidays were defined as any day deemed a federal holiday by the U.S. Government. Weekends were defined as Saturday and Sunday. A composite of holidays, weekends, summer vacation, teacher in-service days, etc. was used to create the variable students out of school. This variable was obtained using the Chicago Public Schools yearly calendar from 2011 to 2012 to the 2016–2017 school year. In the 2012–2013 school year, public schools were split into two different tracks (District Calendar, 2018). Track E reduced the summer vacation but gave extended mid-year breaks while Track R was typical of all previous years. For this analysis, we assumed that all schools followed Track R (charter schools and private schools typically followed the Track R calendar). Month and year were both added as fixed effects to all models. Finally, year was also interpreted independently, with 2012 as the reference, to broadly explore how the number of daily shootings has been changing over time.

### Descriptive analysis

We graphed the average number of shootings per week and daily shootings by daily high temperature. We also graphed for each of the 12 months the average maximum temperature and average number of shootings per day.

### Statistical analysis

To examine the relationship between the exposures of interest and daily number of shootings, we used generalized linear models with a negative binomial distribution (dispersion parameter ranged from 2.3 to 2.9, depending on model) and log link, and the method of generalized estimating equations in R 3.6.0. We built models based on each exposure of interest’s causal structure by identifying a priori those variables that might confound the relationship between each exposure of interest and the daily number of shootings, rather than through statistical fit. This was based off information commonly known about weather patterns (Portmann et al., 2009), including the effect that temperature has on not only the likelihood of precipitation, but also the type (Warmer Air Means More Evaporation and Precipitation, 2017); temperature and speed of wind (Temperature Gradient, n.d.); temperature and level of humidity, and the level of humidity and precipitation (Humidity, n.d.). All variables and their connections were added into a directed acyclic graph (DAG) and the program Dagitty (Textor et al., 2016) to establish the minimal sufficient adjustment sets for each exposure of interest. The minimal sufficient adjustment set includes the minimal number of variables that need to be added to the model in order to prevent bias from the confounders. Because some backdoor, or biasing paths, are blocked by a single confounder, not all confounders are required to be added into the model to get an unbiased result. We were also interested in whether people being outside was associated with a higher rate of shootings. We therefore tested two categorical variables: if students were out of school and a combination of weekends and holidays to determine if there was any multiplicative interaction between these variables and our other variables of interest. If one of these variables was significant, we included it in the model. The minimal sufficient adjustments and any interacting variables were then used to get unconfounded measures of each exposure of interest, which we analyzed via multivariable negative binomial regression at a significance level of 0.05. For expository purposes, estimated coefficients were transformed into changed percentages, such 1.35 becomes a 35% increase.

## Results

### Descriptive

**Fig. 1:**
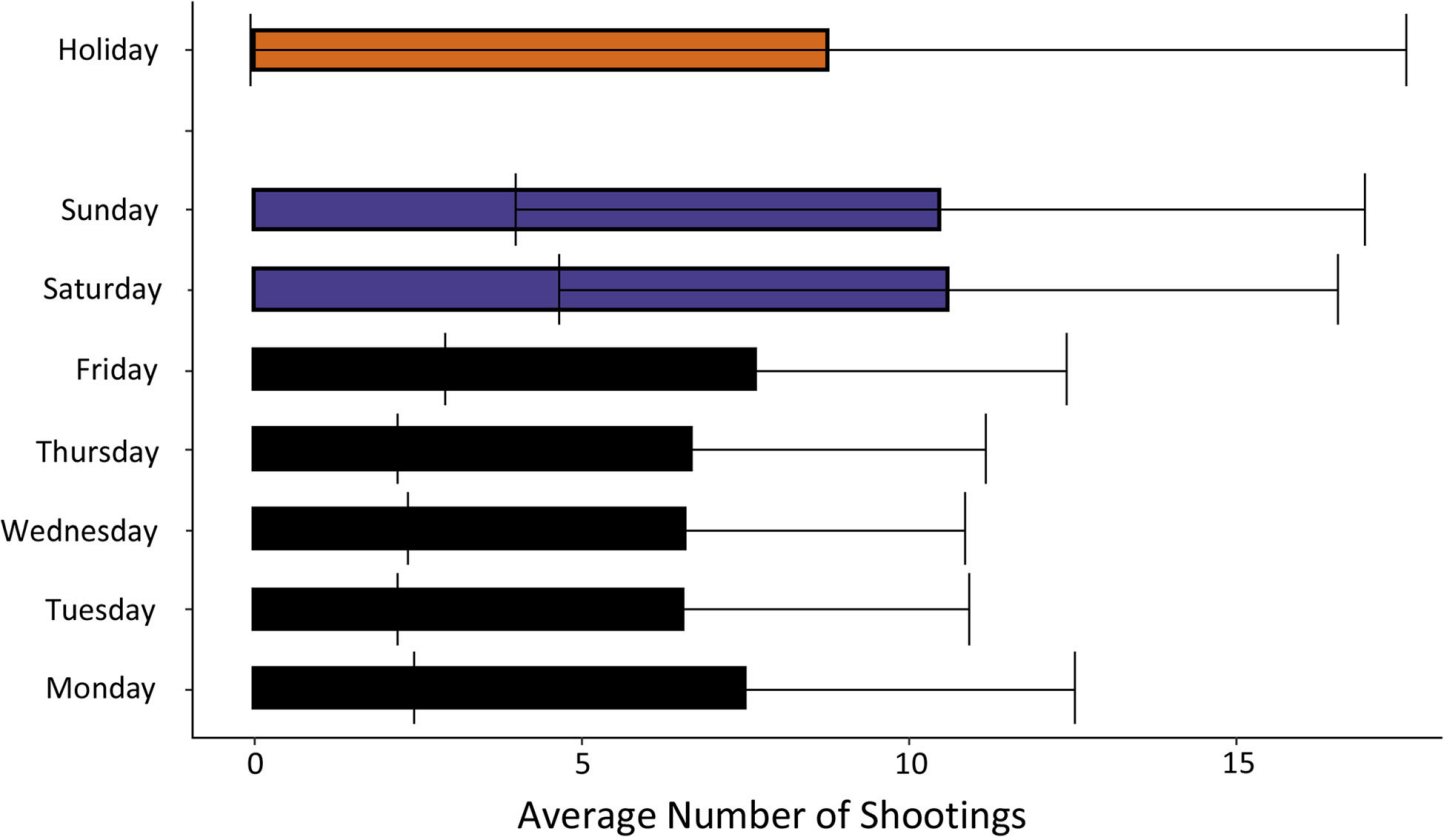
Average number of shootings per day

**Fig. 2:**
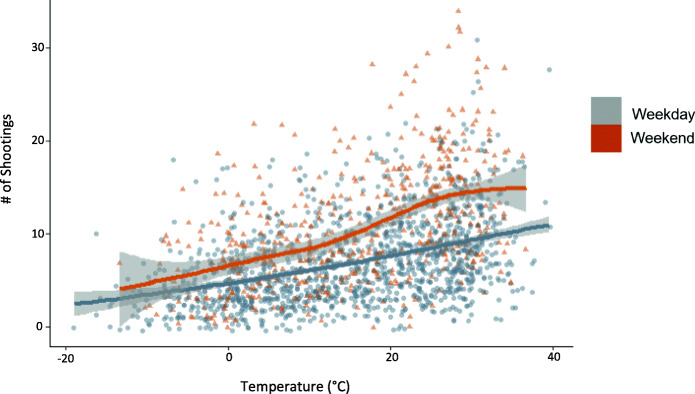
Shootings vs. Temperature

**Fig. 3:**
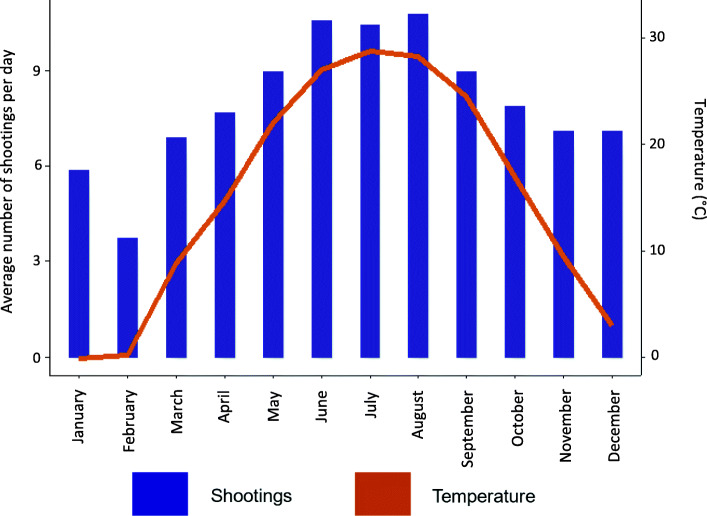
Temperature and shootings by month

From 2012 to 2016, there were a total of 14,633 shooting victims over 1827 days in Chicago—an average of 1 every 3 hours. Descriptive statistics of continuous variables are provided in Table 1. The average high daily temperature was 15.3 °C, approximately a third degree warmer than historical readings. Daily shootings ranged from 0 (31 days) to 34 (on one occasion). The average number of shootings per day of the week and on federal holidays is shown in Fig. 1. Across all days, the mean number of shootings was 8.0. There were on average more shootings on the weekends (> 10) and on holidays (~ 9) than on the weekdays (~ 7). A scatterplot of the number of shooting victims and the daily temperature, stratified by weekend or weekday is shown in Fig. 2. A line of best fit with 95% confidence interval (CI) shows the linear association between temperature and daily number of shootings for both weekdays and weekends. On average, as temperature increased, the number of shootings per day increased, and days with higher levels of shootings are more likely to be on the weekend. Figure 3 shows for each of the 12 months the average temperature and average number of shootings per day. On average, February had the fewest number of average shootings per day while August had the most.

**Table 1:** Descriptive statistics of continuous exposures of interest

	Average	Minimum	Maximum	Standard Deviation
High Temperature (°C)	15.3	−19.9	39.4	12.0
Precipitation Amount (cm)	0.254	0	8.89	0.68
Humidity (Maximum) (%)	85.2	48	100	10.3
Wind (Maximum) (kph)	32.4	11.3	78.9	10.0
Difference in temperature from historical average (°C)	0.28	−18.3	21.1	5.6
**Daily shootings (#)**	**8**	**0**	**34**	**5.4**

### Causal structure

**Fig. 4:**
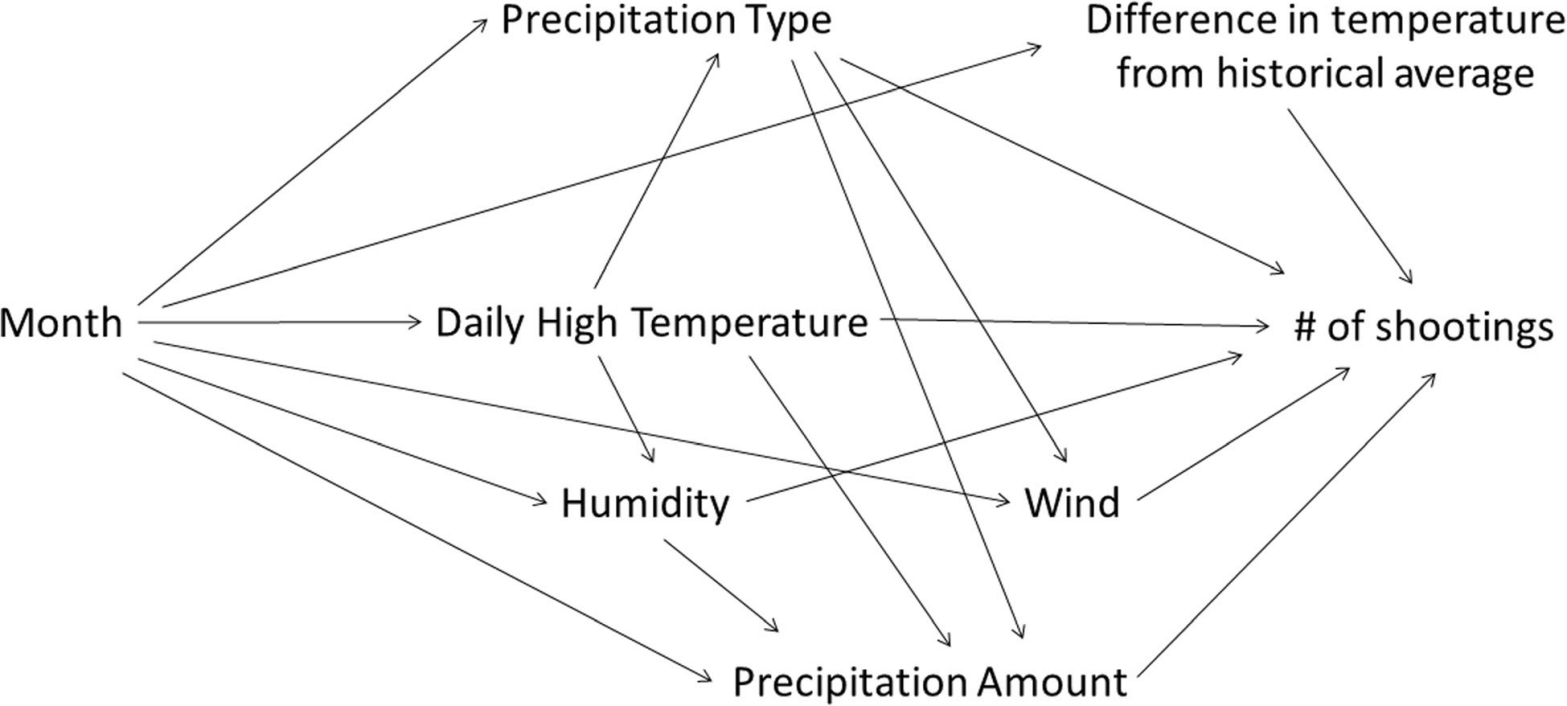
Directed Acyclic Graph (DAG) of variables investigated

The causal structure of the exposure of interests are shown in a directed acyclic graph (DAG) in Fig. 4.

### Causally adjusted models

The adjusted associations--after developing minimal sufficient adjustments for each exposure of interest by identifying confounders and including a fixed effect for month and year-- are shown in Table 2. We found that that the occurrence of a weekend or holiday interacted significantly only with daily high temperature on the multiplicative scale. We did not find that students being out of school was significant with the other covariates for interaction. On weekdays, there was a 34.0% [95%CI (28.1, 40.2] higher rate of shootings per every 10-unit increase in temperature. On weekends and holidays, there was a 41.6% [95%CI: (34.7, 48.9)] higher rate of shootings per every 10-unit increase in temperature. Difference in temperature from historical average, after adjusting for precipitation type, had a similar change in the number of shootings. For every 10-degree higher temperature from the historical average, there were 33.8% more shootings [95%CI (29.1, 39.7)]. No precipitation type was significant after adjusting for temperature; the occurrence of rain reduced shootings (4.4%) but was non-significant (CI -10.4, 1.9). Similarly, precipitation amount was also marginally not significant; for each additional centimeter of precipitation, there was now a − 4.1% decrease in the number of daily shootings [95%CI: (− 9.1, − 0.2)]. Humidity, adjusted for temperature, and wind, adjusted for precipitation type, were not significant in the model. Finally, there seemed to be a modest decrease in daily shootings in Chicago in 2013 compared to 2012, and a modest increase in shootings in 2015, and a large spike in shootings in 2016. There were 73.4% more shootings in 2016 than in 2012 [95%CI: (58.7, 89.6)].

**Table 2:** Associations between weather and shootings in Chicago

Variable	% change in shootings	95%CI	Confounders adjusted for:
Temperature (High)^a^			
*On weekdays*	34.0	(28.1, 40.2)***	n/a
*On weekends/holidays*	41.6	(34.7, 48.9)***	**“**
Δ in temperature from historical average	33.8	(29.1, 39.7)***	Prec. Type
Precipitation Type			Temperature
*Rain*	−4.4	(−10.4, 1.9)	“
*Snow*	0.1	(−9.3, 12.3)	“
*Thunderstorm*	−2.2	(−9.8, 5.3)	“
Precipitation Amount ^b^	−4.1	(−9.1, 0.2)	Prec. Type, Temperature
Humidity (Max)^a^	−0.5	(−3.1, 2.2)	Temperature
Wind (Max)^a^	−1.8	(−4.7, 1.2)	Prec. Type
Year (Ref = 2012)			
*2013*	−13.0	(−4.4, −20.8)**	n/a
*2014*	2.8	(−6.3, 12.7)	n/a
*2015*	18.7	(8.4, 30.0)***	n/a
*2016*	73.4	(58.7, 89.6)***	n/a

^a^for every ten unit increase (°C, %, and km/hr., respectively). ^b^for every 1 cm

* < 0.05, ** < 0.01, *** < 0.001

## Discussion

After adjusting for confounders using the causal structure of the exposures of interest, we found that warmer temperatures and a change in temperature from the historical average were both significantly associated with an increase in shootings in Chicago from 2012 to 2016. Precipitation amount and type (rain) were marginally insignificant and associated with a decrease in the number of shootings. These results are generally supportive of the robust field of literature that has hypothesized that warmer weather is predictive of aggression and violence behavior. (Burke et al., 2015; Baron & Ransberger, 1978; Anderson et al., 2000; Anderson, 1987; Kenrick & MacFarlane, 1986; Cotton, 1986; Field, 1992; Anderson, 1989), including a recently published study that found that individuals were more likely to report bad mental health days when it is warmer outside (Li et al., 2020). However, two unique findings suggest that heat induced aggression is likely not the only explanation for these results. While warmer weather resulted in more shootings, this was more pronounced when there was a weekend or holiday, supporting our hypothesis that people being outside their home might also be a driver of shootings. Furthermore, there were more shootings on days that were warmer than average, including in the colder months. Because some of these “warmer” than average days were relatively mild, and in some cases would be considered cold, this is also not fully explained by the aggression hypothesis and gives credence to the “outdoors” hypothesis. For example, an unusually warm day in January (10 °C) might encourage people to go outside, while the same temperature day in July might be considered cold and result in people not leaving their homes.

There were several differences between our results and those found by Kieltyka et al. (Kieltyka et al., 2016), another study that examined the association between weather and shootings in Chicago. We examined data for 2012 to 2016, a period when gun shootings in Chicago were 36% higher than in 1999–2012 (Kapustin et al., 2017). Kieltyka et al. found an increase in gunshot wounds on Friday and Saturday and not on Sunday. While we did observe an increase in shooting victims on Friday compared to other weekdays, it was still, on average, two fewer shootings per day than on Saturday and Sunday in our data, which corresponds to other existing studies (Michel et al., 2016; Butke & Sheridan, 2010). The differences may be due to changes in the patterns of gun violence and the incompleteness of the Kieltyka data, since individuals who were killed on scene or discharged within 2 hours from the hospital were not included in their data. An additional limitation of their study is that they did not consider the causal structure of their exposures of interest, making the proper interpretation of their results uncertain.

Our findings suggest various types of interventions that might reduce gun violence. For example, gang-related violence-- which typically occurs outside--is the cause or catalyst for most gun deaths in Chicago (Block et al., 1993). One effective policy might be the addition of air conditioning to homes and public places. This would not only improve the quality of life for Chicagoans in the summer, but also might reduce irritably and the need for people to go outside due to excessive heat indoors. Summer work and internships for students, as well as changes to the school schedule could also potentially reduce the number of shootings by keeping adolescents occupied.

Just as knowledge of the micro-locations where shootings are most likely to occur are useful for police policy and planning (Block & Block, 1995; Eck et al., 2005), so too is knowledge of when these events most commonly occur. Indeed, such information should be useful not only to law enforcement, but to any policymakers interested in prevention. We found that Chicago shootings disproportionately occur during extraordinarily warm days, and this is most pronounced during the weekend or holidays. We also found that increased shootings were associated with days that were considerably warmer than historically average, even during the winter. Police may vary the intensity of their patrols accordingly.

Our study has some particular strengths. We believe both the data on daily shootings and on the daily weather are accurate. Our approach also mitigates inference problems that can be caused by including all variables in a single model or when estimates are not properly adjusted for and confounding exists; this helps resolve what is colloquially known as the “Table 2 Fallacy” (Westreich & Greenland, 2013).

Our study has several limitations. First, our study is a cross-sectional, ecological design; however, we make no assumptions about individuals and keep inferences to the level of the environment. Indeed, due to the exposure being inherently environmental, the ecological design could be described as being both obligate and apt for this issue (Susser, 1994). Furthermore, although we consider the causal structure of each exposure of interest, we do not claim the results are necessarily causal due to possible individual level factors that could influence the number of daily shootings—though we believe our estimates to be more accurate than previous studies. Second, we examine daily rather than hourly weather information, and from only a single Troposphere station. Thus, we do not know the weather conditions for exact time of day in the precise communities where shootings occur. For example, precipitation might have only fallen in a specific neighborhood at a specific time, but if it fell by our specific Troposphere station, we effectively assume that all of Chicago experienced that event all day. This misclassification would likely result in an underestimate of the association between type of precipitation. Future studies could include better mapping of weather and shooting incidents through use of GIS technologies by matching the time and place of the shooting to a snapshot of the weather conditions at that moment. Third, for the 2012–2013 school year, we assumed all schools followed the R track. This misclassification also likely resulted in an underestimate of the associations.

## Conclusion

After using a causal structure to adjust for confounders, we found that warmer temperatures, especially on weekends and holidays, and temperatures warmer than the historical average were significantly associated with higher rates of shootings in Chicago from 2012 to 2016. Knowing when events are likely to occur can be as important as knowing where.

## Data Availability

The datasets used and/or analyzed during the current study are available from the corresponding author on reasonable request.
